# Enhancing the Antibacterial Properties of PVDF Membrane by Hydrophilic Surface Modification Using Titanium Dioxide and Silver Nanoparticles

**DOI:** 10.3390/membranes10100289

**Published:** 2020-10-15

**Authors:** Kajeephan Samree, Pen-umpai Srithai, Panaya Kotchaplai, Pumis Thuptimdang, Pisut Painmanakul, Mali Hunsom, Sermpong Sairiam

**Affiliations:** 1 Department of Environmental Science, Faculty of Science, Chulalongkorn University, Bangkok 10330, Thailand; kajeephan0603@gmail.com (K.S.); s.penumpai@gmail.com (P.-u.S.); 2 Institute of Biotechnology and Genetic Engineering, Chulalongkorn University, Bangkok 10330, Thailand; p.kotchaplai@gmail.com; 3 Department of Chemistry, Faculty of Science, Chiang Mai University, Chiang Mai 50200, Thailand; pumis.th@gmail.com; 4 Environmental Science Research Center, Faculty of Science, Chiang Mai University, Chiang Mai 50200, Thailand; 5 Department of Environmental Engineering, Faculty of Engineer, Chulalongkorn University, Bangkok 10300, Thailand; pisut114@hotmail.com; 6 Research Program on Development of Technology and Management Guideline for Green Community, Center of Excellence on Hazardous Substance Management (HSM), Bangkok 10330, Thailand; 7 Research Unit on Technology for Oil Spill and Contamination Management, Chulalongkorn University, Bangkok 10330, Thailand; 8 Academy of Science, The Royal Society of Thailand, Office of the Royal Society, Dusit, Bangkok 10300, Thailand; mhunsom@gmail.com

**Keywords:** polyvinylidene fluoride membrane (PVDF), titanium dioxide nanoparticles (TiO_2_-NP), silver nanoparticles (AgNP), antibacterial property, antifouling property

## Abstract

This work investigates polyvinylidene fluoride (PVDF) membrane modification to enhance its hydrophilicity and antibacterial properties. PVDF membranes were coated with nanoparticles of titanium dioxide (TiO_2_-NP) and silver (AgNP) at different concentrations and coating times and characterized for their porosity, morphology, chemical functional groups and composition changes. The results showed the successfully modified PVDF membranes containing TiO_2_-NP and AgNP on their surfaces. When the coating time was increased from 8 to 24 h, the compositions of Ti and Ag of the modified membranes were increased from 1.39 ± 0.13 to 4.29 ± 0.16 and from 1.03 ± 0.07 to 3.62 ± 0.08, respectively. The water contact angle of the membranes was decreased with increasing the coating time and TiO_2_-NP/AgNP ratio. The surface roughness and permeate fluxes of coated membranes were increased due to increased hydrophilicity. Antimicrobial and antifouling properties were investigated by the reduction of *Escherichia coli* cells and the inhibition of biofilm formation on the membrane surface, respectively. Compared with that of the original PVDF membrane, the modified membranes exhibited antibacterial efficiency up to 94% against *E. coli* cells and inhibition up to 65% of the biofilm mass reduction. The findings showed hydrophilic improvement and an antimicrobial property for possible wastewater treatment without facing the eminent problem of biofouling.

## 1. Introduction

Nowadays, the number of industries is rapidly increasing, thereby posing the risk of serious water pollution. Membrane filtration is among the most popular methods for sustainable wastewater treatment because of its advantages including no phase changes or chemical addition, simple operation, and relatively low energy consumption [1]. Normally, membranes are typically fabricated from hydrophobic materials such as polyvinylidene fluoride (PVDF), polytetrafluoroethylene (PTFE), polyethylene (PE), or polypropylene (PP). Of all the materials used, PVDF is generally applied as a membrane material due to its thermal stability, excellent chemical resistance, and good membrane-forming ability [2]. However, PVDF is a semi-crystalline polymer with repeating units of –CH_2_–CF_2_–, which form hydrophobic structures that can make the membrane more prone to fouling [3,4,5]. Hydrophobic PVDF membrane is susceptible to fouling when the contacting aqueous solution contains hydrophobic species such as protein, resulting in easy absorption on the membrane surface or blockage of the membrane pores leading to decreased permeability [6,7]. Membrane biofouling involves the attachment of microorganisms on the membrane surface to form biofilm, a cluster of cells and their produced extracellular polymeric substances (EPS), which can significantly decrease the separation performance of the membrane [1]. Biofilm and its associated EPS are mainly responsible for the membrane water flux decline since their coverage on the membrane surfaces can block membrane pores and increase water transport resistance [8]. This can shorten the working life of the membrane, increase the operation cost, and finally restrict successful applications. To solve this problem, the membrane hydrophilicity must be increased to induce fouling resistance, thereby preventing the attachment of microbial cells along with the adsorption and deposition of hydrophobic pollutants onto the membrane surface [9].

There have been many attempts at the modification of the hydrophilic layer on the existing membrane surface in order to enhance the hydrophilicity, antibacterial properties and membrane performance, resulting in more efficient membrane applications such as water and wastewater treatment [10,11,12]. One of the most common and effective techniques to increase the hydrophilicity on the membrane surface is the addition of inorganic nano-/micro-particles including SiO_2_, TiO_2_, Al_2_O_3_, and ZrO_2_, which contain an abundance of polar groups on their particle surfaces [12,13]. One of the most popular inorganic particles used is titanium dioxide nanoparticles (TiO_2_-NP) because of its non-toxicity, low cost, and superhydrophilicity [14]. Furthermore, the catalytic activity of TiO_2_-NP was proved to be capable of killing a wide range of microorganisms including endospores, as well as fungi, algae, protozoa, and viruses [15]. For decades, silver nanoparticles (AgNP) have been generally used as an effective broad-spectrum biocide to functionalize filtration membrane surfaces for biofouling mitigation [16,17]. The antibacterial mechanism of AgNP, as reported in previous literature, is the release of silver ions (Ag^+^) that can induce cell membrane damage, promote generation of reactive oxygen species (ROS) and disrupt adenosine triphosphate (ATP) production and DNA replication, ultimately causing the death of bacteria [18]. For the purpose of enhancing the membrane fouling, the changes of membrane properties during the membrane preparation process and surface membrane modification were mainly employed. A previous study has shown that AgNPs deposited on PVDF membrane surface by physical vapor deposition could enhance the antibacterial properties and decrease bacteria growth [19]. However, the modification of PVDF membrane by TiO_2_-NP and AgNP using the dipped coating technique still suffers from a research gap requiring improvement. Also, the contamination of *Escherichia coli* still poses serious public health issues and numerous economic losses [20]. Therefore, TiO_2_-NP/AgNP-modified PVDF membrane could be useful for the treatment of wastewater contaminated with *E. coli*. By using this modification method, the membrane performance could be improved for long term use, thereby offering a more sustainable wastewater treatment technology.

This study aims to enhance the antibacterial and antifouling properties of the PVDF membrane modified by TiO_2_-NP and AgNP using the dipped coating method. The modified membrane was characterized by scanning electron microscopy (SEM) with energy dispersive X-ray spectroscopy (EDS) before being identified for its morphology, contact angle, and porosity. By using *E. coli* as a model strain, the antibacterial property and biofilm formation inhibition of the modified membranes were investigated.

## 2. Methodology

### 2.1. Chemicals and Materials

PVDF flat sheet membrane was purchased from ANOW^®^ (ANOW^®^, Hangzhou, China) with the membrane specifications already reported by the manufacturer (Table 1). A commercial TiO_2_-NP and AgNP were supplied from Prime Nanotechnology (Bangkok, Thailand). The properties of TiO_2_-NP and AgNP as obtained from the manufacturer are summarized in Table 2. Deionized (DI) water was used for solution preparation.

### 2.2. Experimental Procedures

#### Membrane Modification via Titanium Dioxide and Silver Nanoparticles (TiO_2_-NP and AgNP)

**Figure 1:**
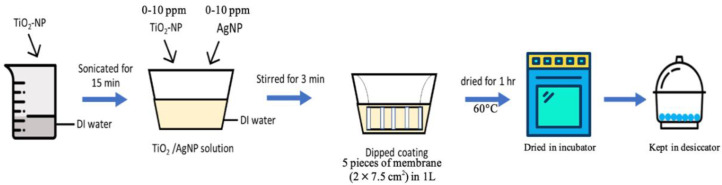
Schematic diagram for TiO_2_-NP/AgNP dipped coating of 75 cm^2^ of flat sheet PVDF membrane into 1L solution.

First, TiO_2_-NP powders were dispersed in 1 L of deionized (DI) water and sonicated for 15 min using an ultrasonic bath (DT100SH, Bandelin, Berlin, Germany) at 320 W, 35 kHz to obtain a homogeneous TiO_2_-NP suspension. Second, AgNP was then added into the solution and the mixture of solution was stirred by magnetic stirrer for 3 min. For membrane modification (Figure 1), 5 pieces of PVDF flat sheet membranes (2 × 7.5 cm^2^) were coated by dipping into the 1 L of nanoparticle suspension (75 cm^2^/L) The coating times (0–24 h) and concentrations of TiO_2_-NP and AgNP (0 and 10 ppm) were varied as shown in Table 3. After that, the modified membranes were incubated in an oven (ULM700, Memmert, Schwabach, Germany) at 60 °C for 1 h to eliminate excess liquid and kept in a desiccator to completely remove moisture before being used for analysis and further experiments.

### 2.3. Membrane Characterization

#### 2.3.1. Contact Angle Measurement

The membrane hydrophilicity was evaluated from the surface contact angle (OCA40, Dataphysics, Filderstadt, Germany). The water contact angle (WCA) of the membrane was measured by the sessile drop method at room temperature. One membrane sample was used for the measurement of each type of membrane (Table 3). DI water was dropped on the top surface of a dried membrane at three different positions on the single piece of membrane, and then the data were presented as a mean of the contact angles with the errors represented as standard deviations.

#### 2.3.2. Morphology and Chemical Composition

Scanning electron microscopy (SEM, JSM-IT500HR, JEOL, Tokyo, Japan) and energy-dispersive X-ray spectroscopy (EDS, JEOL, Tokyo, Japan) were used to investigate the presence of TiO_2_-NP and AgNP as well as the surface morphology of the membrane. For each type of membrane, one membrane sample was used for the analysis. The membranes were cut into pieces and coated with gold under vacuum condition before the observation to avoid the electrostatic charging through a sputter-coater (Balzers UNION Limited, SCD040, Balzers, Liechtenstein). The chemical compositions of the membranes were analyzed three times on the same membrane sample, and the data obtained were presented as a mean with the errors represented as standard deviations. Besides, the outer surface topographies and roughness of the original and modified PVDF membranes were further observed by atomic force microscopy (AFM, SPA 400, SEIKO, Chiba, Japan). The scan area of 10 μm × 10 μm was reported using a tapping mode.

#### 2.3.3. Porosity Analysis

Porosity of the membrane was evaluated using the gravimetric method. Each sample of the membrane (1.5 × 1.5 cm^2^) was weighed using a digital weight balance (MS204S/01, Mettler Toledo, Greifensee, Switzerland) and recorded as the dry weight (m_2_). After that, the membrane was immersed in DI water for 48 h. Then, the sample was weighed immediately and recorded as the wet weight (m_1_). The membrane porosity (ε) was calculated using Equation (1) as follows [21]:

(1) $$\varepsilon =\;\left(\frac{{(m}_{1}-{\;m}_{2}{)/\rho }_{\mathrm{water}}}{\frac{{(m}_{1}-{\;m}_{2})}{\rho_{\mathrm{water}}}\;+\;\frac{m_{2}}{\rho_{p}}}\right)\;\times \;100\%\;$$

where m_1_ is the wet membrane weight (g), m_2_ is the dry membrane weight (g), ρ_water_ is the water density (0.998 g cm^−3^), and ρ_p_ is the PVDF density (1.74 g cm^−3^). The data of the membrane porosity were presented as a mean with standard deviations calculated from three data of three separated membranes.

#### 2.3.4. Permeation Performances

The permeation performances of both original and modified PVDF flat sheet membranes were performed by an in-house dead end filtration device (Sterlitech Corporation, CF042D, Kent, WA, USA). The effective membrane area of a filtration device is 47 cm^2^. For each membrane, the membranes were immersed in DI water for 24 h at room temperature. Prior to measurement, the membrane was pre-compacted at 4 bar for 30 min to obtain the initial flux. DI water was fed into the filtration device at the desired pressure, then, the permeate water obtained at different time intervals was measured using an electronic weighting balance to evaluate the permeate flux (*J*) which was expressed as Equation (2):

(2) $$\;J\;=\;\frac{V}{A\;\times \;t}\;$$

where *V* is the volume of permeate water (*L*), A is the effective area (m^2^) and *t* is the running time (h)

#### 2.3.5. Functional Group Analysis

Raman spectroscopy was carried out in a DXR Raman microscope (Thermo Fisher Scientific Inc., Madison, WI, USA) using a laser excitation wavelength of 780 nm of laser power. The experiments were operated through an aperture of 50-micron slit and a 10×-objective lens with a laser spot of 3.1 µm. Raman spectra were obtained using a 2 s exposure time with 32 accumulations.

### 2.4. Antibacterial Test

#### 2.4.1. *E. Coli* Strains and Growth Condition

*E. coli* were obtained from multiple tube fermentation technique by the standard protocol of the most probable number of coliform organism test (MPN) for gas and acid productions [22]. First, three different volumes of water sample (10, 1 and 0.1 mL) were transferred into each of five Durham tubes containing 10 mL of lactose broth (LB) and incubated at 35 °C for 48 h (ULM 600, Memmert, Schwabach, Germany). The solution from the test tubes showing gas production was transferred to a new tube containing brilliant green lactose bile broth (BGLB) and incubated overnight at 35 °C. Then, 0.1 mL of the BGLB was spread on nutrient agar (NA) plate before incubated at 37 °C for 24 h. The isolated colonies, identified as *E. coli*, were further restreaked onto an NA plate until a single colony is obtained. Once purified, the isolates were maintained in liquid nutrient broth (NB) at 4 °C. This suspension was used for antibacterial and biofilm inhibition tests.

#### 2.4.2. Antibacterial Test

The experiment was modified from Liu, Yao, Ren, Zhao, and Yuan (2018) [1]. First, the membrane (1.5 × 1.5 cm^2^) was immersed into the suspension of *E. coli* cells (pre-adjusted with liquid NB to obtain the initial concentration of 10^6^ CFU/mL) with NB in 24 well-plates. Then, the well plates were incubated at 37 °C for 48 h. The membranes were rinsed gently with 0.85% NaCl twice to remove the excess *E. coli* suspensions and non-adhered cells. The membranes were then transferred into sterile test tubes containing 10 mL of 0.85% NaCl before vortex mixing for 1 min to detach the cells. Finally, 0.1 mL of cell suspension was spread on NA plate and incubated overnight at 37 °C. The number of viable cells on the membrane was expressed as colony forming unit (CFU/mL). Data were obtained from three wells, each of which contained a single piece of membrane, and represented as a mean and standard deviations.

#### 2.4.3. Biofilm Inhibition Test

The biofilm inhibition test was conducted to determine the performance of the modified membranes to inhibit biofilm formation on surface by measuring the biomass of biofilm. The pre-weighed membranes (1.5 × 1.5 cm^2^) were immersed into the suspension of *E. coli* cells in a similar manner to the antibacterial test, but the incubation time was increased to 72 h to allow biofilm formation. The membranes were rinsed with DI water to remove the loosely bound cells. The membranes were oven-dried at 60 °C for 1 h followed by air-drying. The weight of the membrane was measured by a digital weight balance (UMX2, Mettler Toledo, Greifensee, Switzerland). Biofilm mass was determined by the difference between the weight of the membrane before and after being immersed into the suspension of *E. coli* cells [23]. Data were obtained from three different pieces of membrane, and represented as a mean and standard deviations.

## 3. Results and Discussion

### 3.1. Hydrophilic Membrane Modification

#### 3.1.1. Water Contact Angle (WCA)

(a) Effect of coating time

**Figure 2:**
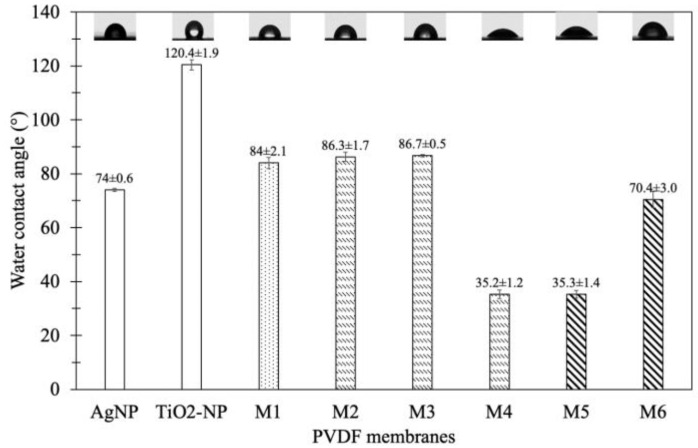
The water contact angle (WCA) of M1 (original), M2 (8 h), M3 (16 h), M4 (24 h) at mixed 10 ppm TiO_2_-NP/10 ppm AgNP, and M5 (10 ppm TiO_2_-NP/20 ppm AgNP) and M6 (20 ppm TiO_2_-NP/10 ppm AgNP) at coating time of 24 h.

WCA was measured to evaluate the membrane hydrophilicity that plays a significant role in membrane permeability [24]. The results showed that the WCAs of the original PVDF membrane M1 and the modified membrane M2, M3, and M4 were 84.0° ± 2.1°, 86.3° ± 1.7°, 86.7° ± 0.5° and 35.2° ± 1.6°, respectively (Figure 2). The WCA of M4 was decreased to be lower than that of M2 and M3 due to higher TiO_2_-NP and AgNP content. This could result from the longest coating time for M4, which allows more TiO_2_-NP/AgNP to attach on the membrane surface as shown in SEM results in the previous section (Figure 2). Furthermore, polyetherethersulfone incorporated with AgNP exhibited the increased hydrophilicity, leading to the increase of water permeability associated with higher affinity [25]. This was because of high specific surface area and fraction of nanoparticles that provide specific functionality to the modified membrane for facilitating the hydrophilicity and permeability. Therefore, the results indicated that the coating time of 24 h can improve the membrane hydrophilicity.

(b) Effect of TiO_2_-NP/AgNP concentration

The results of WCAs of the original membrane (M1) and TiO_2_-NP/AgNP modified membranes (M4–M6) by varied nanoparticle concentrations were illustrated in Figure 2. The WCA of M1 was 84.0° ± 2.1° due to the hydrophobic property of the original membrane. As TiO_2_-NP/AgNP were introduced into the membranes, the WCA were decreased to 35.2° ± 1.6° (M4), 35.3° ± 1.4° (M5) and 70.4° ± 3.0° (M6) for the nanoparticle ratios of 10 ppm TiO_2_-NP/10 ppm AgNP, 10 ppm TiO_2_-NP/20 ppm AgNP and 20 ppm TiO_2_-NP/10 ppm AgNP, respectively, implying more hydrophilicity. Figure 2 also shows that the WCA of AgNP membrane (control) was decreased to 74.0° ± 0.6°. Since AgNP has high specific surface area and high fraction of surface atom, the adhesion of AgNP on the surface can alter the membrane properties to be more hydrophilic and permeable [25]. On the other hand, the WCA of TiO_2_-NP membrane (control) was increased to 120.4° ± 1.9°. The differences in contact angle between the control TiO_2_-NP and AgNP membranes suggested that the optimal ratio between TiO_2_-NP and AgNP contents could play an important role in the coating and differentiation of WCA. As the contents of TiO_2_-NP/AgNP were increased to 10 ppm TiO_2_-NP/20 ppm AgNP and 20 ppm TiO_2_-NP/10 ppm AgNP for M4 and M5, their WCAs were similarly decreased. This could be due to the agglomeration of TiO_2_-NP and AgNP from high concentrations; therefore, the WCAs were not decreased even though the AgNP concentration was increased. Moreover, M6 was coated by highest concentration of TiO_2_-NP compared to M4 and M5, but the WCA was slightly decreased compared to the original membrane M1 (Figure 2), which could also result from the excess of TiO_2_-NP on the membrane to cause TiO_2_-NP agglomeration. According to the results, increasing TiO_2_-NP and AgNP concentrations did not decrease the WCA; therefore, using 10 ppm TiO_2_-NP/10 ppm AgNP for M4 coating was adequate to improve the hydrophilicity of the membrane.

#### 3.1.2. Membrane Morphology

(a) Effect of coating time

**Figure 3:**
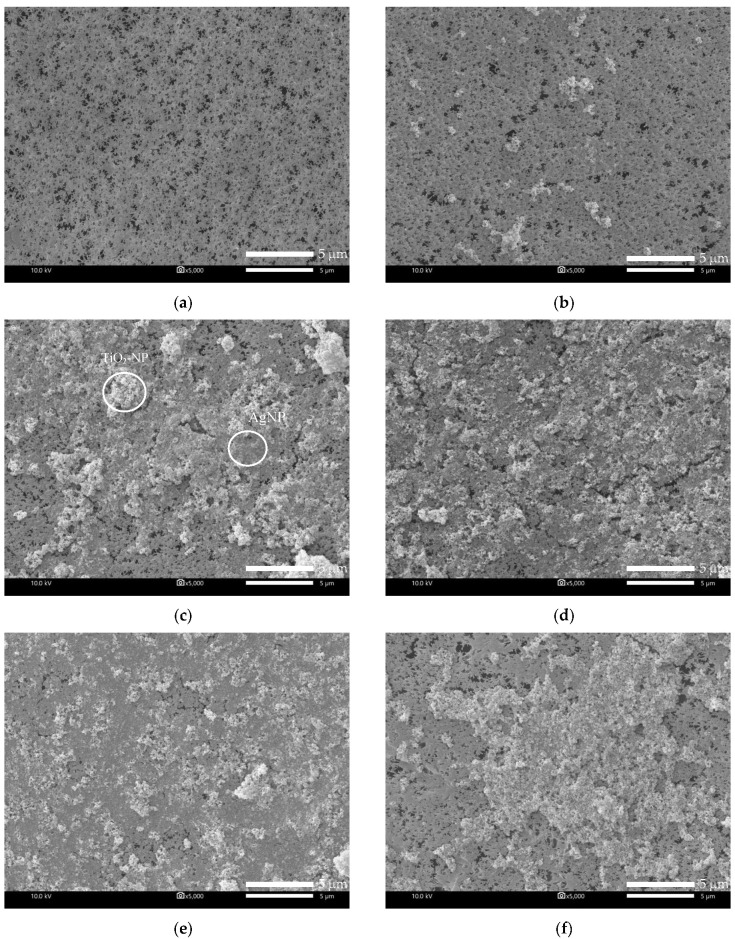
Scanning electron microscope (SEM) images of the membranes modified by different coating times: (**a**) M1 (original); (**b**) M2 (8 h); (**c**) M3 (16 h); (**d**) M4 (24 h) at mixed 10 ppm TiO_2_-NP/10 ppm AgNP; (**e**) M5 (10 ppm TiO_2_-NP/20 ppm AgNP); and (**f**) M6 (20 ppm TiO_2_-NP/10 ppm AgNP) at coating time of 24 h (magnification ×5000).

**Figure 4:**
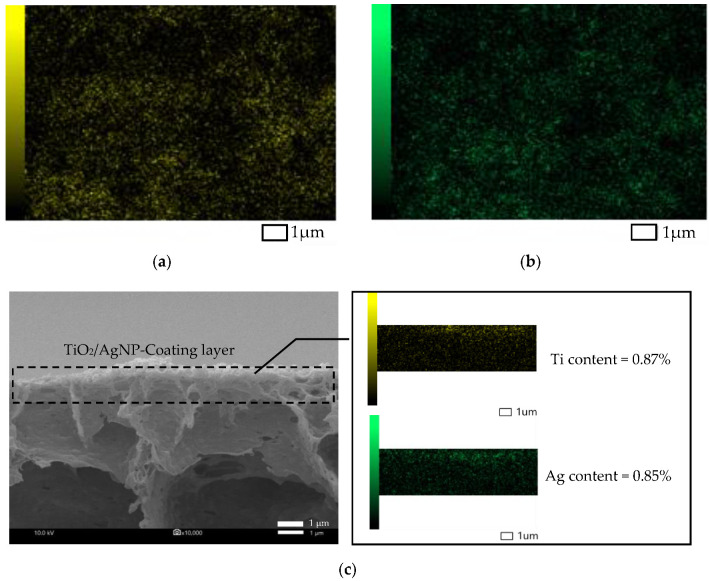
Mapping of particle distribution of modified membrane with 10 ppm TiO_2_ and 10 ppm AgNP for 24 h (M4): (**a**) TiO_2_-NP on membrane surface; (**b**) AgNP on membrane surface; and (**c**) TiO_2_-NP and AgNP of X-section (magnification ×10,000).

The PVDF membrane was immersed in the solution of 10 ppm TiO_2_-NP and 10 ppm AgNP for varied periods of time to study the effect of coating time on the membrane. The results showed that, compared with the original membrane (Figure 3a), the TiO_2_-NP/AgNP was observed on the modified membranes (Figure 3b–d), suggesting the successful coating. More particles of TiO_2_-NP/AgNP were found on the membrane surface when the coating time was increased from 8 to 24 h for M2 (Figure 3b) and M4 (Figure 3d), respectively. Figure 3b shows that the agglomerated TiO_2_-NP was distributed throughout the surface while AgNP was hardly spotted; however, as the coating time increased, TiO_2_-NP and AgNP were both clearly dispersed and observed on membrane M3 (Figure 3c) and M4 (Figure 3d). In addition, TiO_2_-NP and AgNP were mostly attached together because these nanoparticles have high surface energy that makes them tend to agglomerate to reach more stable state [26]. EDS mapping of TiO_2_-NP and AgNP distributions on M4 were also carried out and presented in Figure 4. It can be seen clearly that the distributions of TiO_2_-NP and AgNP were observed on the membrane surface after coating of 10 ppm TiO_2_-NP and 10 ppm AgNP for 24 h as illustrated in Figure 4a–c. These results supported the successful coating and the layer of TiO_2_-NP and AgNP could improve the hydrophilicity and possibly increase the membrane permeate flux.

Additionally, elemental compositions of the modified membranes were detected by EDS. Three elements including Ti, O, and Ag were analyzed to confirm the existence of TiO_2_-NP and AgNP from the membrane modification, and the results are shown in Table 4. From the results, the modified membranes M2–M4 contain more Ti than Ag even though the same concentrations of TiO_2_-NP and AgNP were used, which indicates that Ti might adhere on the membrane surface better than Ag. Furthermore, the percentage of O was higher than that of Ti in all membranes due to the elemental ratio of TiO_2_. Table 4 also confirms the results in Figure 2 that the number of TiO_2_/AgNP that adhered on the membrane surface depends on the coating time. Table 4 shows the composition of Ti, O and Ag on M4 which was lower than M3 (coating time of 24 h). This might be due to more agglomeration of TiO_2_-NP/AgNP on M4 compared with M3, which decreases the surface area of TiO_2_-NP/AgNP on the membrane. These results indicated that TiO_2_-NP/AgNP were highly adhered by using the coating time of 16 h (membrane M3).

(b) Effect of TiO_2_-NP/AgNP concentration

The morphology of the modified membranes using different nanoparticle concentrations, which were 10 ppm TiO_2_-NP/10 ppm AgNP (M4), 10 ppm TiO_2_-NP/20ppm AgNP (M5) and 20 ppm TiO_2_-NP/10 ppm AgNP (M6), are shown in Figure 3. The smoother surface of the original PVDF membrane (M1) showed that there are no particles on the surface compared with that of the modified membranes, in which a large number of TiO_2_-NP/AgNP can be observed. When the concentrations of TiO_2_-NP and AgNP increased, higher distribution of the nanoparticles on the membrane surface can be observed (Figure 3 e–f). Likewise, Yang, Peng, Wang, and Liu (2010) reported that high content of TiO_2_ concentration motivates the aggregation phenomenon on the membrane surface [27]. Furthermore, the presence of TiO_2_-NP and AgNP on the membranes was confirmed by EDS as reported in Table 4. The results show that the amounts of Ti and O adhered on M6 were twice as much as M4 and M5 due to higher TiO_2_-NP concentration (20 ppm) used for M6. From the results of WCA (Figure 2) and the TiO_2_-NP and AgNP distribution mappings in Figure 4, it was evident that the WCA was positively correlated to TiO_2_-NP and AgNP content on membrane surface. It could be concluded that the deposited TiO_2_-NP and AgNP on membrane surface would play important role for enhancing hydrophilicity.

#### 3.1.3. Surface Roughness

(a) Effect of coating time

**Figure 5:**
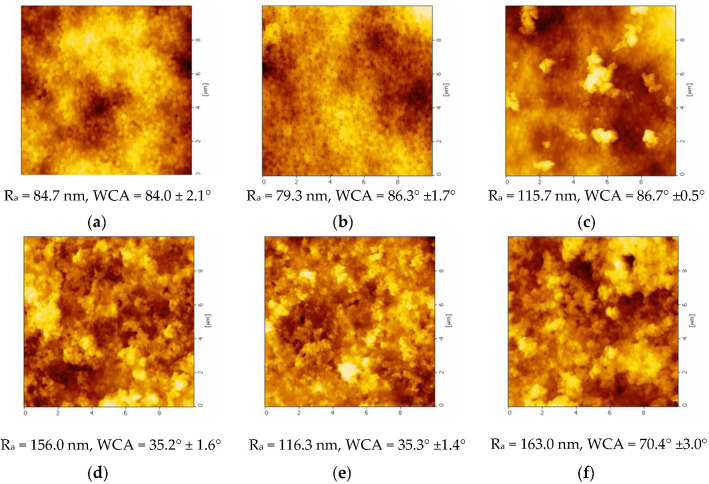
Atomic force microscopy (AFM) image topography of membranes: (**a**) M1 (original); (**b**) M2 (8 h); (**c**) M3 (16 h); (**d**) M4 (24 h) at mixed 10 ppm TiO_2_-NP/10 ppm AgNP; (**e**) M5 (10 ppm TiO_2_-NP/20 ppm AgNP); and (**f**) M6 (20 ppm TiO_2_-NP/10 ppm AgNP) at coating time of 24 h.

The AFM images of the membrane coated with the solution of 10 ppm TiO_2_-NP and 10 ppm AgNP at different times (8–24 h) are shown in Figure 5. As observed, the original PVDF membrane (M1) exhibits a smooth surface area with the mean surface roughness (R_a_) value of 84.7 nm while M2, M3, and M4 membranes show rougher surface at 79.3, 115.7, and 156.0 nm, respectively, with the increase of coating time. The increase of coating time leads to more agglomeration of TiO_2_-NP/AgNP particles on the membrane, which could dramatically enhance the surface roughness and, therefore, efficiently increase hydrophilicity. The lower WCA obtained after TiO_2_-NP/AgNP coating on membrane surface could be due to the enhance surface roughness. According to the Wenzel’s model, the wettability of surface is amplified by increasing the surface roughness; in other words, an increase in surface roughness leads to the increase of surface hydrophilicity [7,28,29].

(b) Effect of TiO_2_-NP/AgNP concentration

According to Figure 5, the surface roughness of PVDF flat sheet membrane was also affected by the addition of TiO_2_-NP/AgNP. The AFM images show that the surface of the membranes is rougher compared with the original PVDF membrane like presented in SEM images. R_a_ of M5 and M6 was 116.3 and 163.0 nm, respectively. R_a_ of the modified membrane was increased when the TiO_2_-NP/AgNP concentration was increased from 10 ppm to 20 ppm. There is a difference of high peak and low valley with increasing the TiO_2_-NP/AgNP concentration. The increase of high peaks leads to higher surface roughness. This could explain the lower contact angle of modified membrane compared with the original membrane, showing higher surface roughness.

#### 3.1.4. Porosity

(a) Effect of coating time

The porosities of the original PVDF membrane and the membranes modified by varying coating time are reported in Table 5 and calculated by Eq.1. The results showed that increasing the coating time slightly reduced the porosity of the membrane. The porosity was increased from 61.33% for the original membrane M1 to 67.19%, 67.18%, and 66.74% for M2, M3, and M4, with the coating time of 8 h, 16 h and 24 h, respectively. This could be explained by the fact that longer coating time resulted in more TiO_2_-NP and AgNP agglomerations, which increased the opportunity for large particles to block the membrane pores.

(b) Effect of TiO_2_-NP/AgNP concentration

The porosities of the original PVDF membrane and membranes modified by varying TiO_2_-NP/AgNP concentrations were shown in Table 5. The modified membranes with the nanoparticle concentrations of 10 ppm TiO_2_-NP/10 ppm AgNP (M4), 10 ppm TiO_2_-NP/20 ppm AgNP (M5), and 20 ppm TiO_2_-NP/10 ppm AgNP (M6) provided the increased porosities of 66.74% ± 2.45%, 66.45% ± 2.78% and 64.10% ± 4.73%, respectively, compared with 61.33% ± 2.83% of the original PVDF membrane (M1). Previous study has also found that the addition of nanoparticles on the membrane could increase the porosity [30]. However, among the modified membranes, the porosity was decreased when the content of the nanoparticles increased, which might be due to the blockage of membrane pores by the aggregation of nanoparticles [9]. A study found that the addition of 4 wt% TiO_2_-NP in combination with PVDF and sulfonated polyethgersulfone (PES) exhibited the reduction of water permeability owing to the decreased porosities [31]. In addition, TiO_2_-NP could induce the extremely rapid precipitation and produce a thick dense top layer that can also block the membrane pores [32]. The results indicated that the modified membranes with TiO_2_-NP and AgNP were improved in their porosity, whereas increasing the TiO_2_-NP/AgNP concentrations did not induce any porosity improvement.

#### 3.1.5. Pure Water Flux of Membranes

**Figure 6:**
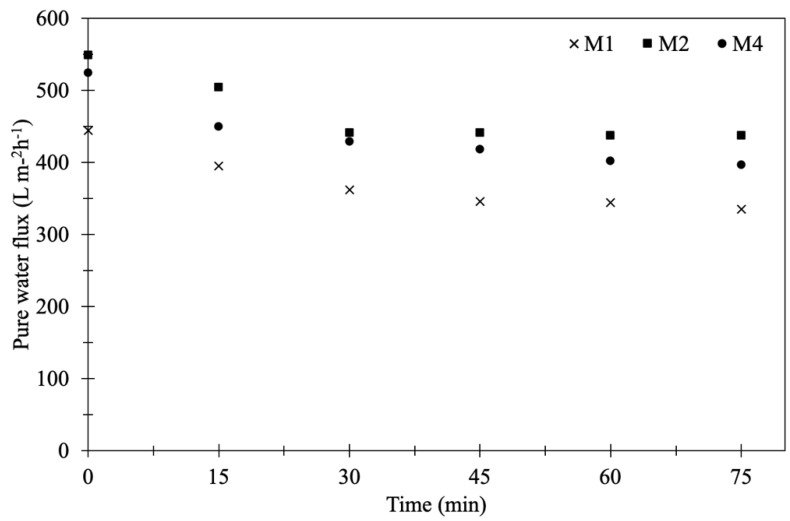
Water fluxes of original PVDF membrane and modified membranes (M1, M2 and M4).

Pure water flux of the original and modified PVDF flat sheet membranes was measured at different time intervals under 4 bar of pressure to evaluate the water permeability. Figure 6 exhibits the initial pure water flux (*t* = 0, after setting for 30 min) and the results showed that the modified PVDF membrane coated with TiO_2_-NP and AgNP gave higher flux than that of the original PVDF membrane. The original membrane (M1) gave the lowest initial flux at 444.7 L m^−2^ h^−1^ while the M2 and M4 provided the fluxes up to 548.8 and 524.1 L m^−2^ h^−1^, respectively. The coating time of TiO_2_-NP and AgNP affected the pure water flux to a greater extent. The presence of hydroxyl functional group from TiO_2_-NP and AgNP contributed to improve the hydrophilicity, thereby improving the pure water flux. M4 had the lowest contact angle (more hydrophilic) but the initial flux was lower than that of M2. This was because the coating time of 24 h (M4) could allow more TiO_2_-NP and AgNP accumulation on the membrane surface and membrane pores, resulting in the increased membrane thickness and then lower water flux compared to 8 h (M2).

**Figure 7:**
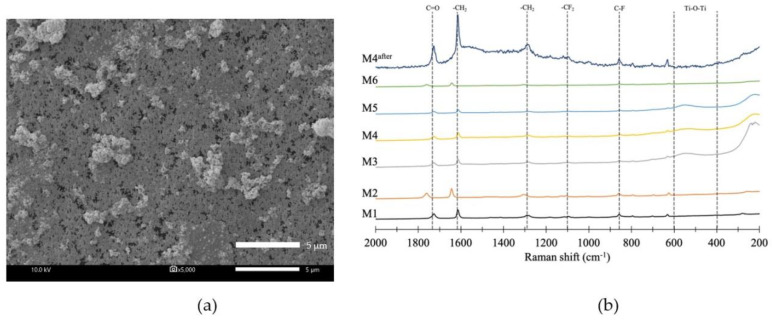
Morphology and functional groups of membrane after pure water flux testing: (**a**) SEM image of M4; and (**b**) Raman spectra of membrane.

In order to verify the stability and durability of the nanoparticles coated on the PVDF membrane, the original and modified PVDF membranes were tested by water filtration for the period of 75 min as illustrated in Figure 6. The stable permeate flux of M2 and M4 was higher than that of M1, which was still more than 400 L m^−2^ h^−1^. The flux decline was observed after 30 min and then it was not significant. To determine the durability of TiO_2_-NP and AgNP layer coated on membrane surface after 75 min water filtration, SEM-EDS of M4 was again measured together with Raman analysis and the result showed that both TiO_2_-NP and AgNP were still presented on the membrane surface. However, the Ti and Ag concentrations before and after the pure water flux test were decreased from 4.29% to 1.01% and 3.62% to 0.55%, respectively, as shown in Table 4. It was clear that the amount of Ti and Ag might be lost with water, suggesting inadequate stability and durability of nanoparticles on the membrane due to lack of strong chemical bonding on the membrane surface. Figure 7a shows the SEM images of M4 after water filtration, which also confirmed the presence of TiO_2_-NP and AgNP. Raman spectroscopy confirmed that TiO_2_ was strongly deposited on modified PVDF membrane. The results of original and modified membranes exhibited a number of adsorption bands at 1703 cm^−1^, 1650 cm^−1^, 1453 cm^−1^, 1100 cm^−1^, and 840 cm^−1^, which were attributed to C=O, CH_2_, vibration of CH_2_, CF_2_, CF stretching vibration [33,34,35], respectively. For the modified membrane, a broad band from 600 to 400 cm^−1^ was associated with the Ti-O-Ti group and the peak of 443 cm^−1^ could be assigned to Ti-O-Ti stretching vibration [34]. No difference was observed after Raman shift at 2200 cm^−1^. The results of Raman analysis along with SEM-EDS confirmed the deposition of TiO_2_-NP on PVDF membrane before and after testing. To improve the durability and retention of nanoparticles on the membrane, the stabilization of those nanoparticles on membrane surface by chemical/plasma activation and crosslinked with polylactic acid might be an effective method to provide the TiO_2_-NP and AgNP layer stability on membrane surface.

### 3.2. Antibacterial Properties of Modified Membrane

#### 3.2.1. Antibacterial Test

(a) Effect of TiO_2_-NP/AgNP concentration

Both TiO_2_-NP and AgNP are well-known for their antimicrobial properties [36]. TiO_2_-NP can generate reactive oxygen species (ROS), especially under ultraviolet (UV) irradiation, damaging cellular components, e.g., lipid membrane, protein, or DNA [37]. Besides oxidative stress, the released Ag^+^ could inactivate several enzymes and interact with DNA, resulting in cell death [38]. In this study, the PVDF membranes coated with TiO_2_-NP and AgNP were tested for their antibacterial property to reduce the number of *E. coli* cells.

**Figure 8:**
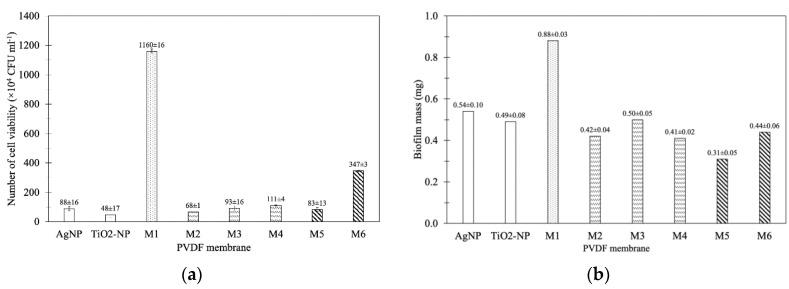
Antibacterial properties and biofilm inhibition test of membranes: (**a**) the number of viable *E. coli* cells and (**b**) biofilm formation on membranes.

The number of viable *E. coli* cells adhered onto PVDF membrane were 1.16 × 10^7^ CFU mL^−1^. In this study, the modified membranes showed antibacterial properties under dark condition that the modification of PVDF membrane with either 10 ppm of TiO_2_-NP or AgNP significantly decreased the number of viable cells on the membrane to 0.48 × 10^6^ and 0.89 × 10^6^ CFU mL^−1^, respectively (Figure 8a). In comparison with M1, the presence of both TiO_2_-NP and AgNP (M4) significantly decreased the number of viable cells on the membrane by 90% (1.11 × 10^6^ CFU mL^−1^) (*p*-value > 0.5), comparable to those coated with only TiO_2_-NP or AgNP. Increasing the amount of AgNP to 20 ppm (M5, 0.83 × 10^6^ CFU mL^−1^) did not significantly affect the antibacterial property of the TiO_2_-NP/AgNP dip-coated PVDF membrane (M4) (*p*-value > 0.5). This is possibly due to the similar chemical composition between M4 and M5 (Table 4). On the contrary, a higher amount of TiO_2_-NP (M6, 3.46 × 10^6^ CFU mL^−1^) substantially reduced the antibacterial activity of the TiO_2_-NP/AgNP dip-coated PVDF membrane. The particular antibacterial efficiency depends on their size, shape, and surface areas that would release free radicals for the inactivation of bacterial cells [39]. Even though the higher content of Ti and Ag was detected on the M6 membrane (Table 4), too high a TiO_2_-NP concentration led to the agglomeration onto PVDF membrane (Figure 3f). This could reduce the contact area between bacterial cells and nanoparticles thus decreasing the antibacterial activity. Similar results have been observed by Soo et al. (2020) in *Salmonella* Albany LCUM0022 and *Bacillus cereus* LCUM0001 under contact with Ag dopant with TiO_2_ that reduces the microbial growth toward bacterial cell growth inhibition [40]. *E. coli* cell growth was also decreased with the addition of catalyst under dark condition [41]. This was the permeation of active TiO_2_-NP into bacterial cell during the dark condition, resulting in the agglomeration and attachment at the bacterial membrane [42]. Of all the modified membranes, the results indicated that the mixed solution of 10 ppm TiO_2_-NP and 10 ppm AgNP (M4) was suitable for the antibacterial property.

(b) Effect of coating time.

The number of *E. coli* cells on the PVDF membranes modified with 10 ppm TiO_2_-NP/10 ppm AgNP at various coating times are also presented in Figure 8b. The dipped coating of 10 ppm TiO_2_-NP/10 ppm AgNP onto the PVDF membrane for 8 h (M2), 16 h (M3) and 24 h (M4), reduced the number of adhered cells by 94%, 92%, and 90%, respectively. Longer coating time resulted in the lower antibacterial efficiency of the modified membrane even though higher Ti and Ag components were observed (Table 4). This likely caused by the agglomeration of nanoparticles on the modified membrane (Figure 3); therefore, M2 with lower agglomeration could contain more nanoparticle surface area to allow more interaction sites between particles and cells, thereby showing higher antibacterial property than M3 and M4.

#### 3.2.2. Biofilm Inhibition Test

(a) Effect of TiO_2_-NP/AgNP concentration

The effect of nanoparticles-modified membrane on inhibition of biofilm formation, or antifouling property, was evaluated by using the gravimetric method. While 0.88 ± 0.03 mg of biofilm mass was detected in the unmodified PVDF membrane (M1), the coating of either TiO_2_-NP or AgNP could significantly reduce the amount of biofilm to 0.49 ± 0.08 and 0.54 ± 0.10 mg, respectively (Figure 8b). The results are in agreement with the antibacterial property (Figure 8a). The coating of both nanoparticles (M4) decreased the amount of biofilm mass to 0.41 ± 0.02 mg, corresponding to 53% reduction. In comparison with M4, the increase of AgNP to 10 ppm TiO_2_-NP/20 ppm AgNP (M5) further reduced the biofilm mass by 65% (0.31 ± 0.05 mg). On the other hand, the higher amount of TiO_2_-NP in M6 did not improve the antifouling property (50% reduction of biomass). However, compared to either TiO_2_-NP or AgNP-modified membrane, the coating of both TiO_2_-NP or AgNP did not increase the antibacterial property (Figure 8a), yet the synergistic effect on the antifouling property was observed (Figure 8b). Besides the inactivation of bacterial cells, the altered surface hydrophilicity also affects the biofilm formation, i.e., antifouling property of the modified membrane. Ayyaru and Ahn (2018) [32] reported that increasing the hydrophilicity of the membrane surface could increase antifouling properties. Fouling or the formation of biofilm is initiated by the attachment of cells to the surface using the associated organelles such as flagellar and pilli [43]. Once the cells irreversibly attached to the surface, they started to produce EPS substances such as carbohydrates and proteins to form biofilm structure, which could completely block the membrane pores at this stage [44]. It has been proposed that the hydration layer on the hydrophilic surface reduces the adhesion of protein to the surface, resulting in the lower attached cells for biofilm formation [45].

(b) Effect of coating time

Since the antifouling property of PVDF membrane coated with both TiO_2_-NP and AgNP (M4) was more effective than those coated with only TiO_2_-NP or AgNP (Figure 8b), the effect of coating time on TiO_2_-NP/AgNP-modified membrane was investigated. The biofilm mass of M2, M3, and M4 were 0.42 ± 0.04, 0.50 ± 0.05 and 0.41 ± 0.02 mg, respectively compared to M1 (0.8751 ± 0.03) as illustrated in Figure 8b. The PVDF membrane modified with 10 ppm TiO_2_-NP/10 ppm AgNP reduced biofilm formation by 43–53%. Increasing the coating time (8, 16, and 24 h) did not affect the antifouling property of the modified membrane since the biofilm mass of M2, M3 and M4 were not significantly different (*p* ˃ 0.05). It should be noted that increasing the coating time also decreased the WCA in this study (Figure 2). Even though it has been found that lower WCA led to more hydrophilic membrane that could inhibit microorganisms adhering to the membrane [46], similar results of the modified membranes in this study suggested that more factors could play a role in antifouling properties.

## 4. Conclusions

In this work, the modified PVDF membrane by TiO_2_-NP/AgNP dipped coating technique was developed to improve the hydrophilicity of the membrane along with its antibacterial and antifouling properties. The results of SEM and EDS showed that the amount of TiO_2_-NP/AgNP on the membrane surface increased with longer coating time and higher TiO_2_-NP/AgNP concentrations. Furthermore, increasing the coating time and TiO_2_-NP/AgNP concentrations could decrease the WCAs of the modified membranes. Compared with the porosity of 61.33% ± 2.83% of the original PVDF membrane, the porosity of the modified membranes could be increased up to 66.74% ± 2.45% using the coating time of 24 h. At this fixed coating time, increasing the nanoparticle concentrations to 20 ppm of TiO_2_-NP and 20 ppm of AgNP could increase the porosities to 64.10% ± 4.73% and 66.45% ± 2.78%, respectively. Noteworthy antibacterial and antifouling properties of the TiO_2_-NP/AgNP-modified membrane were reported. The modified membranes could reduce the number of adhered *E. coli* cells by approximately 90% and the biofilm formation by at least 43%. The PVDF membrane dip-coated in 10 ppm TiO_2_-NP and 10 ppm AgNP for 8 h showed the highest antibacterial (94% inactivation) and antifouling properties (65% reduction); the increased TiO_2_-NP or AgNP content or coating time did not improve these properties due to the agglomeration of nanoparticles. This modified membrane is a promising alternative for improving membrane-based wastewater treatment.

## Figures and Tables

**Table 1:** Specifications of the polyvinylidene fluoride (PVDF) membrane.

Pore Size (µm)	0.45
Membrane Porosity (%)	80
Thickness (µm)	110
Color	White

**Table 2:** Properties of titanium dioxide and silver nanoparticles (TiO_2_-NP and AgNP).

Properties	TiO_2_-NP	AgNP
Appearance	White Powder	Yellow Brown Colloid
Crystalline Structure	80% Anatase, 20% Rutile	-
Primary Particle Size (nm)	21	-
Average Particle Size (nm)	-	5–20
Particle Shape	-	Nanospheres
	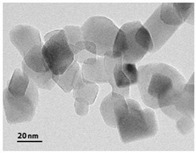	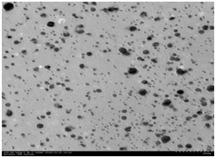
pH	-	6 ± 1
Specific gravity (g mL^−1^)	-	1.01
Tamped density (g L^−1^)	130	-
Specific surface area (m^2^ g^−1^)	50	-
Content (wt%)	˃99.5	-

**Table 3:** Concentrations of TiO_2_-NP and AgNP and coating time for membrane modification.

Membrane	Concentration of TiO_2_-NP (ppm)	Concentration of AgNP (ppm)	Immersion Time (h)
Original PVDF			
M1	0	0	0
Control PVDF			
TiO_2_-NP	10	0	24
AgNP	0	10	24
Modified PVDF			
M2	10	10	8
M3	10	10	16
M4	10	10	24
M5	10	20	24
M6	20	10	24

**Table 4:** Chemical compositions of the modified membranes. (The standard deviation (S.D.) was obtained from 1 sample of each condition).

Membrane	C (%)	F (%)	O (%)	Ti (%)	Ag (%)
M1	55.11 ± 0.18	43.61 ± 0.22	1.28 ± 0.08	-	-
M2	58.16 ± 0.21	35.05 ± 0.28	4.36 ± 0.14	1.39 ± 0.13	1.03 ± 0.07
M3	35.89 ± 0.17	30.42 ± 0.34	20.51 ± 0.27	8.33 ± 0.22	4.84 ± 0.11
M4	45.02 ± 0.17	34.42 ± 0.27	12.65 ± 0.18	4.29 ± 0.16	3.62 ± 0.08
M4^after^	55.62 ± 0.07	37. 59 ± 0.11	5. 36 ± 0.06	0.93 ± 0.02	0.51 ± 0.01
M5	46.57 ± 0.18	33.61 ± 0.30	12.19 ± 0.20	4.06 ± 0.16	3.57 ± 0.09
M6	30.87 ± 0.16	25.46 ± 0.31	24.48 ± 0.30	13.80 ± 0.26	5.39 ± 0.11

Remark: (1) M4^after^ is the chemical compositions of membrane after testing with pure water flux. (2) S.D. is varied between different elements and analytical lines and for the same element in different matrices.

**Table 5:** Porosity of the membranes.

Membrane	Porosity (%)
M1	61.33 ± 2.83
M2	67.19 ± 3.70
M3	67.81 ± 3.79
M4	66.74 ± 2.45
M5	66.45 ± 2.78
M6	64.10 ± 4.73
TiO_2_-NP (control)	65.99 ± 10.35
AgNP (control)	66.52 ± 3.35
